# Motion Artifact Quantification and Sensor Fusion for Unobtrusive Health Monitoring

**DOI:** 10.3390/s18010038

**Published:** 2017-12-25

**Authors:** Christoph Hoog Antink, Florian Schulz, Steffen Leonhardt, Marian Walter

**Affiliations:** Philips Chair for Medical Information Technology, RWTH Aachen University, 52074 Aachen, Germany; florian.schulz@rwth-aachen.de (F.S.); leonhardt@hia.rwth-aachen.de (S.L.); walter@hia.rwth-aachen.de (M.W.)

**Keywords:** motion artifacts, unobtrusive sensing, sensor fusion, motion capture, heart rate, medical signal processing, biosignals, ambient assisted living

## Abstract

Sensors integrated into objects of everyday life potentially allow unobtrusive health monitoring at home. However, since the coupling of sensors and subject is not as well-defined as compared to a clinical setting, the signal quality is much more variable and can be disturbed significantly by motion artifacts. One way of tackling this challenge is the combined evaluation of multiple channels via sensor fusion. For robust and accurate sensor fusion, analyzing the influence of motion on different modalities is crucial. In this work, a multimodal sensor setup integrated into an armchair is presented that combines capacitively coupled electrocardiography, reflective photoplethysmography, two high-frequency impedance sensors and two types of ballistocardiography sensors. To quantify motion artifacts, a motion protocol performed by healthy volunteers is recorded with a motion capture system, and reference sensors perform cardiorespiratory monitoring. The shape-based signal-to-noise ratio SNR${}_{S}$ is introduced and used to quantify the effect on motion on different sensing modalities. Based on this analysis, an optimal combination of sensors and fusion methodology is developed and evaluated. Using the proposed approach, beat-to-beat heart-rate is estimated with a coverage of 99.5% and a mean absolute error of 7.9 ms on 425 min of data from seven volunteers in a proof-of-concept measurement scenario.

## 1. Introduction

Not so long ago, monitoring of vital signs was almost exclusively performed in clinical environments. However, this has changed dramatically with the advancements in microelectronics and the ubiquitous availability of computational power in the last years. Today, technologies that could only be found in the hospital some decades ago are now available in smartphones, smartwatches, body sensor networks, and objects of everyday life [1,2,3]. These novel sensor technologies could lead to tremendous breakthroughs in domestic health monitoring applications. However, traditional technologies are applied by experts in a well-defined environment. This is not the case in a home application scenario, where user and patient are the same person. Moreover, coupling between subject and sensor is often volatile and a priori unknown. The result is a varying signal quality, which can be influenced by disturbances due to, for example, motion. As of today, a variety of algorithms for dealing with these influences exist. In particular, the fusion of multiple estimators applied to one sensor [4], data fusion of multiple sensors of the same modality [5] as well as fusing multimodal signals [6] has shown to increase robustness and accuracy.

While most body sensor networks include a variety of sensing principles, many research groups in the area of unobtrusive monitoring with integrated sensors still focus rather on individual modalities than on multimodal sensor setups. However, exceptions exist, and a general trend towards multimodal setups can be observed. The works by Wartzek et al. describe a sensor design that integrates capacitively coupled electrocardiography (cECG) with through-cloth reflective photoplethysmography (PPG) in one compact sensor element [7]. In addition to this fusion on the hardware level, a robust fusion algorithm has been described that increased coverage when both modalities are jointly analyzed. In [8], Baek et al. presented a notable multimodal setup, the “smart health monitoring chair”, equipped with cECG, through-cloth reflective PPG and ballistocardiography (BCG). In addition to beat-to-beat intervals, pulse arrival time was estimated and shown to correlate well with blood pressure measurements in a group of five healthy volunteers. Similarly, the “Sensing Chair” [9] was equipped with sensors to acquire ECG, pulse wave and body weight.

One major problem of all unobtrusive sensing modalities are motion artifacts. It can also be noted that most of the published works focus on the detection and removal of noise and artifacts and not on their quantification and modeling, and those models that exist usually describe the impact on individual modalities. For example, the influence of motion on cECG via triboelectricity was analyzed in [10]. In [11], the effect of motion artifacts on cECG was reduced using an injection signal. Similarly, flexible electrodes [12] or arrays of capacitive ECG sensors [13] were used to improve robustness towards motion artifacts. In the field of (unobtrusive) PPG acquisition, several approaches have been presented to detect and reduce the influence of motion artifacts, ranging from statistical approaches [14,15] over adaptive filters [16,17] to Kalman smoothers [18] and independent component analysis [19]. To compensate motion artifacts in BCG measurements, threshold-based methods [20] and additional noise references have been proposed [21].

To the best of our knowledge, no setup exists that can reproduce or quantify the effects of motion artifacts on simultaneously and unobtrusively recorded cardiorespiratory signals. We hypothesize that this constitutes a severe obstacle in the field of unobtrusive monitoring, as it impedes both the determination of an optimal combination of sensors and the development of sensor fusion algorithms that could otherwise exploit respective model information. In this work, several contributions to alleviate the problem are presented.

First, an armchair termed “MuSeSe” (“**Mu**lti**Se**nsor**Se**ssel” in German) is equipped with several sensing modalities to monitor cardiorespiratory activity, including cECG, reflective PPG, two types of BCG sensing principles and high-frequency (HF) impedance sensors. For reference, standard ECG and respiratory flow is recorded.

To address the problem of reproducible motion artifacts, several solutions seem feasible: As a first step, recordings could be performed where subjects were asked to perform a certain movement protocol, which would be manually documented or annotated in the recorded data. This could help to gain first insights into the influence of motion artifacts and could be used to evaluate artifact detectors. However, this would give no quantitative information about the amplitude of the respective movement and may not report to which degree the subject actually followed the protocol. Another method to create reproducible artifacts would be to use some sort of mechanical setup to induce predefined movements. The two major problems of such an approach are complexity and realism of biosignals. On the one hand, a sturdy mechanical setup capable of generating large forces would be required to displace a human subject accurately, and thus safety becomes a major concern. On the other hand, a simpler setup might be only capable of displacing some sort of small signal-generating phantom. While individual modalities might be reproduced accurately, a phantom that can change its electrical, mechanical and optical properties in a way that it mimics multimodal cardiorespiratory signals realistically is hard to conceive. Thus, in this work, a third option is explored. Measurements are performed with nine healthy volunteers that are instructed to perform a motion protocol that covers a wide range of realistic movements. Moreover, the subjects are instructed to move with two different amplitudes. The aforementioned reference sensors for cardiorespiratory activity are included in the setup, and thus the sensor signals that are acquired unobtrusively can be compared to a gold standard. The remaining problem of movement quantification is solved by including a motion capture system into the setup, thus allowing the quantification of the subject’s movement with sub-millimeter accuracy. As a consequence, the exact adherence to the protocol is unnecessary, as any deviation can be measured and quantified.

Finally, based on the results of the motion artifact analysis, the system’s performance is evaluated in a setup showcasing a potential home monitoring scenario. Here, seven healthy volunteers were asked to watch a video of their choice, during which reference and unobtrusive vital sign monitoring was performed.

The multisensor armchair and the proposed motion protocol were presented as a preliminary result at the “40th Annual International Conference of the IEEE Engineering in Medicine and Biology Society” [22].

## 2. Methods

**Figure 1:**
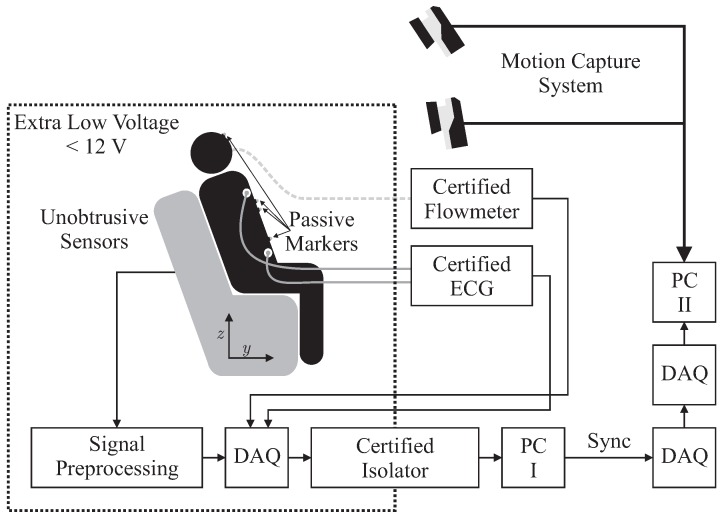
Overall measurement setup. The test subject is only connected to certified reference sensors and unobtrusive sensors that are isolated using certified power supplies and isolators.

In this section, the physical setup including unobtrusive and reference sensors, the measurement protocol and the methods of evaluation are described. Figure 1 presents the overall measurement scenario.

### 2.1. Hardware Setup

Several sensors were integrated into the backrest and the seat of an armchair for unobtrusive biosignal acquisition. To ensure safety, all sensors were operated with extra low voltage (below 12 V), which was provided by an EN 60601-1 certified power supply. After analog to digital conversion with a “NI USB-6212” (National Instruments, Austin, TX, USA) data acquisition system (DAQ) at a sampling frequency of $f_{s}=100$ Hz, data was transferred to PC I via USB using an EN 60601-1 certified USB isolation device. For reference biosignal acquisition, an “MP30” patient monitor, manufactured by Philips, Amsterdam, The Netherlands and a “Pneumotach Amplifier 1 Series 1110” by Hans Rudolph, Inc., Shawnee, KS, USA were used as analog front ends. To record reference motion data, the “Oqus” system from Qualisys AB, Göteborg, Sweden was used. It was configured with seven “Opus 500+” infrared (IR) tracking cameras with a resolution of four megapixels and a maximum frame rate of $f_{s,\max}=180$ Hz. The aperture of the C-mount lenses with a focal length of 13 mm was set to f/4.0 and manual focusing was performed. Illumination was provided by rings of IR LEDs integrated into each camera unit. Seven passive reflective markers were used and tracked at $f_{s,M}=100$ Hz on PC II. To record analog data synchronously with marker positions, a synchronized USB-DAQ could be accessed by the Oqus system. However, since no isolation in accordance with EN 60601-1 could be guaranteed, the aforementioned method for sensor data acquisition by PC I was used. In addition, a synchronization signal was generated using a second DAQ, which was in turn recorded by the synchronized DAQ connected to PC II.

**Figure 2:**
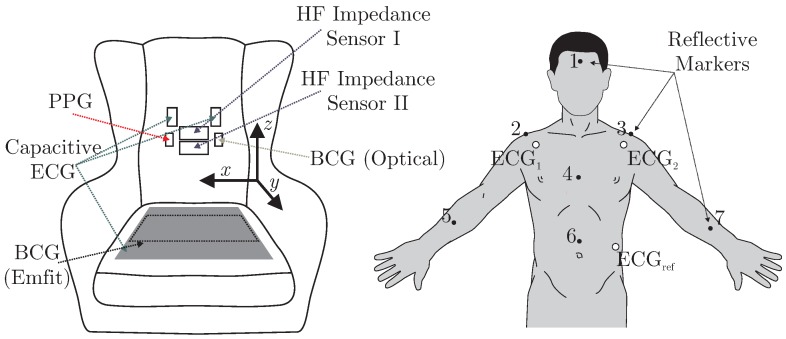
(**left:**) schematic of the armchair and placement of sensors, the dashed rectangular on the seat shows the placing of the EMFi sensor, while the gray rectangular illustrated the DRL electrode of the capacitive ECG. The arrows indicate the coordinate system; (**right:**) position of tracking markers and reference ECG electrodes. Modified from ([22], Figure 1) © 2017 IEEE.

**Figure 3:**
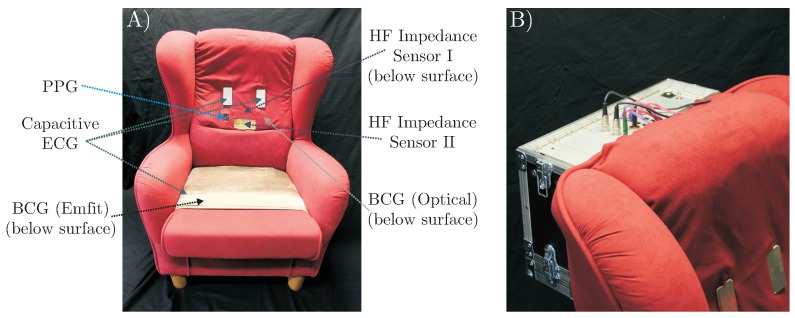
(**A**) photograph of “MuSeSe”; (**B**) closeup of the case containing analog and digital front-ends as well as the DAQ.

A total of six different sensors for unobtrusive biosignal acquisition were integrated into the backrest and the seat of the armchair (see Figure 2 (left)). As the focus of this work is the overall system, no in-depth descriptions and schematics of the individual sensing modalities are given. The interested reader is referred to the respective references for more details (see below). For the acquisition of cardiac-related signals, four modalities were included. For one, a capacitive ECG system as described in [23] with two electrodes of ultra-high input impedance was integrated into the backrest. A driven-right-leg (DRL) electrode was placed on the seat using conductive fabric. To acquire a PPG signal, a sensor consisting of three NIR LEDs and a centered photodiode was implemented using the design proposed in [7]. The signal of the photodiode was bandpass-filtered (0.15 to 5 Hz) and amplified by 40 dB. An optical BCG (BCG Opt) was recorded with a second PPG module that was not facing the subject, but the padding of the backrest as described in [5]. Another BCG signal was recorded from the seat of the chair using an “EMFi transducer” mat (L-Series by *Emfit Ltd., Vaajakoski, Finland*). A custom built analog amplifier/bandpass with a passband of 0.01 to 200 Hz and gain of 24.61 dB was used. To record respiratory signals, two capacitive HF impedance sensors were used with a straightforward approach: the thorax is modeled as a resistor-capacitor (RC)-element whose parameters change with cardiorespiratory activity. This RC-element is part of an oscillator circuit, and thus cardiorespiratory activity can be estimated by monitoring the resonant frequency [24,25,26]. A photograph of the complete setup is shown in Figure 3.

### 2.2. Measurement Protocol

To evaluate the multimodal sensor system, two different measurement scenarios were investigated: “Motion Sequence” and “Video Sequence”. All recordings were performed on healthy volunteers in our lab as self-experimentation, and informed consent was obtained prior to our measurements. Details are given in Table 1, including age, height, body-mass-index (BMI) and gender. Mean values and standard deviation (SD) are calculated. Note that subjects were free to participate in either scenario and in both scenarios.

The first scenario aimed at identifying the effect of various movements on unobtrusive biosignal acquisition. Every 30 s, the subjects were asked to perform one of 16 specific movements as listed in Table 2.

In the first half, movements (column 2) were performed with a low amplitude (column 3), in the second half, a higher amplitude was asked for (column 4). Between movements, subjects were asked so sit as still as possible. The recording started and ended with 60 s of motionless sitting. A total of 81 min of data from nine different subjects, including 5994 heartbeats, was recorded.

The second set of measurements were performed to analyze the system in a realistic application scenario. For this, subjects were asked to sit in the armchair and watch a movie of their choice. A total of 425 min of data from seven subjects, including 28,555 heartbeats, was recorded. The data is publicly available as part of the UnoViS database [27], accessible for free from https://www.medit.hia.rwth-aachen.de/unovis/.

### 2.3. Performance Analyses and Information Fusion

Using the “Opus 500+” tracking software, the position of each marker was exported as a three-dimensional coordinate. In the calibration process, the origin and orientation of the coordinate system was set as displayed in Figure 2. To analyze the motion, the root mean square (RMS) value of displacement was analyzed for each coordinate separately. Since each marker position is given with respect to the origin of the coordinate system, we define movement as the AC component of each coordinate. Thus, for a signal with *N* samples, the RMS movement values were calculated via

(1) $$\begin{array}{cc} {\mathrm{RMS}}_{x}=\mathrm{RMS}\left[x^{\prime }\right]= & \sqrt{\frac{1}{N}\sum_{n=0}^{N-1}{\left(x\left[n\right]-\bar{x}\right)}^{2}}, \end{array}$$

(2) $$\begin{array}{cc} {\mathrm{RMS}}_{y}=\mathrm{RMS}\left[y^{\prime }\right]= & \sqrt{\frac{1}{N}\sum_{n=0}^{N-1}{\left(y\left[n\right]-\bar{y}\right)}^{2}}, \end{array}$$

(3) $$\begin{array}{cc} {\mathrm{RMS}}_{z}=\mathrm{RMS}\left[z^{\prime }\right]= & \sqrt{\frac{1}{N}\sum_{n=0}^{N-1}{\left(z\left[n\right]-\bar{z}\right)}^{2}}, \end{array}$$

with $\bar{x},\bar{y},$ and $\bar{z}$ being the respective mean values, i.e., the DC component of that coordinate. For each instruction in the protocol, the motion was quantified and averaged over all subjects. To focus the movement estimation on the thorax, markers one (head), five (right arm) and seven (left arm) were excluded and motion estimation was averaged over the remaining markers.

To analyze the effect of motion on the different modalities, three evaluations were performed. First, the effect of motion on the cardiac signal quality was analyzed. In many technical applications, the signal-to-noise ratio (SNR) is used for this type of analysis. However, a definition of SNR for biomedical signals is hard to obtain. One straightforward approach analyzes the frequency domain content of a cardiac signal and defines energy below a certain threshold (for example 5 Hz) as related to the desired signal and energy above as related to noise. While this can be implemented easily, it is based on the assumption that the influence of noise and artifacts is dominant in the high-frequency range. If this was the case, a perfect SNR could be achieved with low-pass filtering. Moreover, the sensors used in this setup all contained analog bandpass filters and thus a frequency-domain SNR analysis would report a high SNR even if the sensors were measuring no cardiac signal at all.

**Figure 4:**
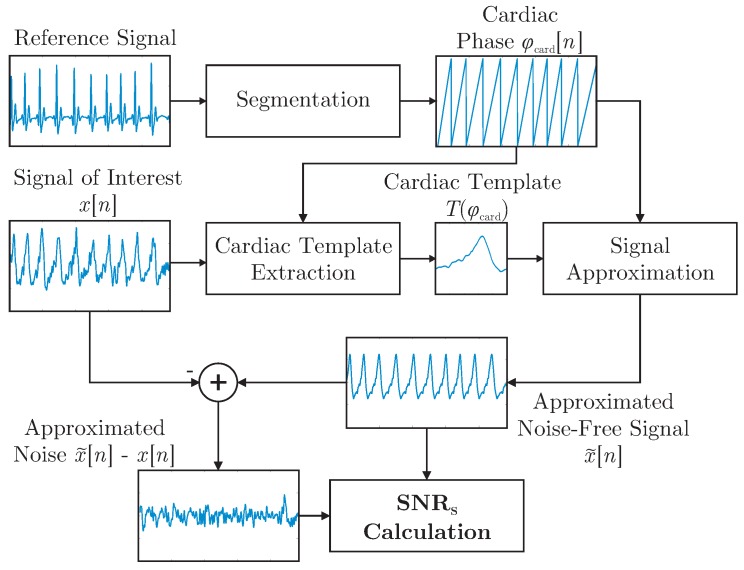
Overview of the ${\mathrm{SNR}}_{S}$ approach. Note that $T_{\mathrm{card}}\left(\varphi_{\mathrm{card}}\right)$ is obtained during an artifact-free episode of x[n] (visualized here). Once obtained, it can be used to analyze the signal of interest anytime a reference segmentation is available (see also Figure 5).

**Figure 5:**
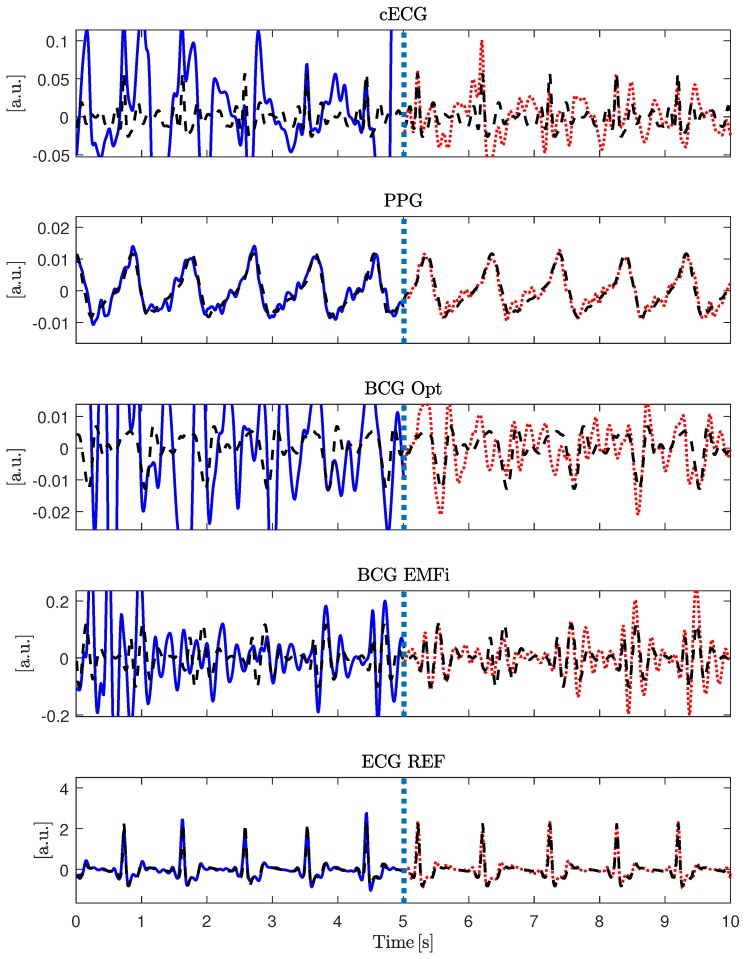
Visualization of the signal approximation step of the ${\mathrm{SNR}}_{S}$ calculation for all modalities. The dashed vertical line separates the artifact segment from the artifact free period. The solid blue line represents the signal recorded during movement, the red pointed line after the movement has stopped. The black dashed line represents the approximation of the signal based on the extracted template and the reference segmentation. Note that, for this specific example, ECG REF and PPG are almost unaffected by the motion.

It was demonstrated in [28] that a template function represented in terms of the cardiac phase $\varphi_{\mathrm{card}}\left[n\right]$ can be used to approximate arbitrary cardiac-related signals. In this work, the concept was expanded and used to define the shape-based SNR, ${\mathrm{SNR}}_{S}$, which is visualized in Figure 4. First, R-peaks detected in the reference ECG were used to segment the signal of interested x[n] into individual cardiac cycles represented in terms of a cardiac phase $\varphi_{\mathrm{card}}$. In essence, all cycles were then interpolated to be of unit length and averaged to obtain the template $T_{\mathrm{card}}\left(\varphi_{\mathrm{card}}\right)$. For each modality and for each episode of the motion protocol, a separate template was extracted. Each template was based on the artifact free period right after the respective motion. This was done instead of creating a single template based on *all* artifact free episodes to account for changes in signal morphology that can result from a change in posture after motion. To estimate ${\mathrm{SNR}}_{S}$, the reference ECG and the extracted templates were used to create an estimation of the noise-free signal $\tilde{x}\left[n\right]$ via interpolation of $T_{\mathrm{card}}$ during both the artifact-free and the artifact period. The process is visualized for the artifact-free scenario in Figure 4. Figure 5 gives an example of the signal approximation for all modalities with and without artifacts.

To calculate ${\mathrm{SNR}}_{S}$, a calculation in analogy to the regular SNR was used:

(4) $$\begin{array}{c} {\mathrm{SNR}}_{S}\left(x\right)=10\log\frac{\sum_{n=0}^{N-1}{\left(\tilde{x}\left[n\right]\right)}^{2}}{\sum_{n=0}^{N-1}{\left(\tilde{x}\left[n\right]-x\left[n\right]\right)}^{2}}\;\left[\mathrm{dB}\right]. \end{array}$$

Here, *N* is the length of the signal x[n], and $\tilde{x}\left[n\right]$ is the signal’s noise-free estimation using the segmentation obtained from the reference ECG and the extracted template. To quantify the dependency of ${\mathrm{SNR}}_{S}$ on the motion amplitudes ${\mathrm{RMS}}_{x,y,z}$, the correlation coefficient ρ was calculated for each sensor and coordinate axis.

In the second experiment, a more application-oriented method of evaluation was used: in [6], an augmentation to an algorithm termed xCLIE (multi-channel extended continuous local interval estimator) for beat-to-beat interval estimation [4] was presented, which incorporates information from previous intervals to improve the current estimation. It was demonstrated that the algorithm can perform sensor fusion of multimodal cardiorespiratory signals to increase both coverage and accuracy. The interested reader is referred to [4,6] for details on the algorithm. In short, self-similarity of consecutive heart-beats is exploited for interval estimation. Thus, no a priori information about the signal’s morphology has to be available, which makes the algorithm modality-independent. To check the signal’s reliability, the algorithm calculates the ratio of the peak to the area under the curve of an autocorrelation-type function in a moving window. If this ratio is below a user-defined, fixed threshold, the algorithm deems the segment too noisy and reports no beat/interval. Thus, in addition to accuracy in terms of estimation error, the algorithm needs to be evaluated in terms of coverage, i.e., the ratio of detected beats $n_{\mathrm{xCLIE}}$ to the reference annotations $n_{\mathrm{ref}}$,

(5) $$\begin{array}{c} \mathrm{Coverage}=\frac{n_{\mathrm{xCLIE}}}{n_{\mathrm{ref}}}\cdot 100\%. \end{array}$$

To analyze the effect of motion quantitatively, the estimation error and the coverage were analyzed for each modality, when fusing all modalities using the original algorithm and when using the adaptive prior introduced in [6]. Evaluation was performed in terms of absolute interval estimation error ($e_{\mathrm{abs}}$) in milliseconds and coverage in percent.

**Figure 6:**
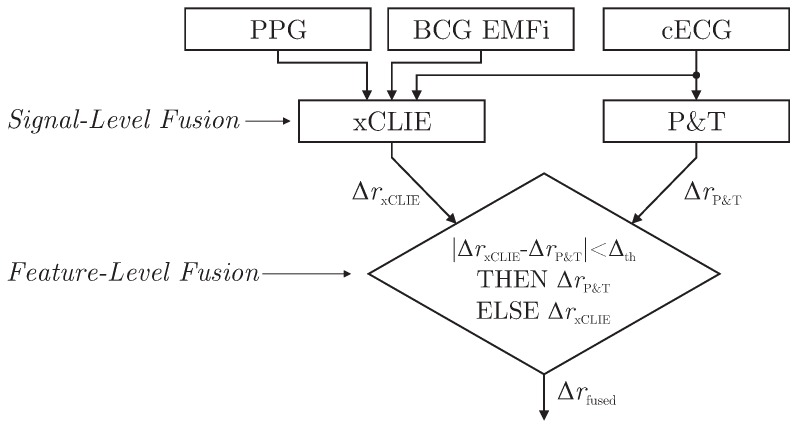
Combined signal and feature-level fusion approach to improve beat-to-beat interval estimation.

As demonstrated in [6], the xCLIE algorithm is a powerful tool to estimate beat-to-beat intervals from multimodal sources. Moreover, the fusion of multiple channels was shown to increase both accuracy and robustness. In particular, the capacitively coupled ECG is known to be disturbed easily by motion artifacts due to triboelectric [10] and other effects [11]. On the other hand, with the exception of cECG, all cardiac-related signals recorded by the sensors of the chair are based on mechanical effects. Thus, even under perfect measurement conditions, estimated intervals would differ from those derived from the reference ECG if the mechanical pulse changed its position or shape relative to the QRS complex due to physiological effects. Thus, a mixed-level fusion algorithm was proposed as demonstrated in Figure 6.

The xCLIE algorithm was used to estimate intervals $\Delta r_{\mathrm{xCLIE}}$, fusing cardiac-related signal on the signal level. In addition, an implementation of the Pan and Tompkins (P&T) algorithm [29] was used to localize R-peaks in the capacitive ECG and derive the respective intervals $\Delta r_{P}\&T$. Next, a straightforward decision rule performed fusion on the feature level: if an interval derived from cECG via QRS-detection was available and the absolute difference of both estimators was smaller than a heuristically determined threshold $\Delta_{\mathrm{th}}$, the ECG-derived interval $\Delta r_{P}\&T$ was chosen for $\Delta r_{\mathrm{fused}}$. Otherwise, $\Delta r_{\mathrm{xCLIE}}$ was used.

## 3. Results

In the following, the results are presented. First, the general functionality of the hardware setup is evaluated qualitatively. Next, quantitative results for the motion sequence and the video sequence are reported.

### 3.1. Measurement Hardware

**Figure 7:**
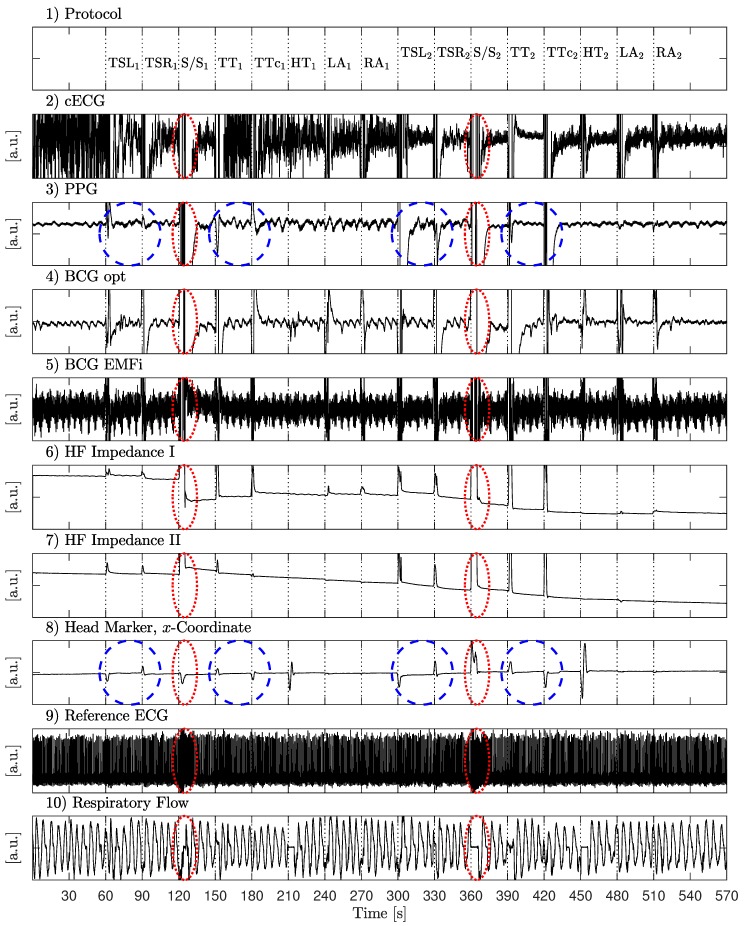
Measurement results for subject 2. Row 1 shows the protocol, rows 2 to 7 display the raw data of the unobtrusive sensors, rows 8 to 10 show the reference signals. Stand up/sit down is marked with red (pointed) ellipsoids in all channels. The blue (dashed) ellipsoids highlight the low and high amplitude torso shifts and torsions in the motion reference and the PPG channel. Note the different effects of left/right shift in the PPG channel. Modified from ([22], Figure 4) © 2015 IEEE.

In Figure 7, the waveforms of the six unobtrusive sensors, the motion protocol, the *x*-coordinate of marker one (head), and the reference sensor output are shown for one subject. Several observations can be made.

First, the motion protocol changes in marker position and resulting artifacts in the unobtrusive sensing signals were well synchronized. As expected, the stand up/sit down maneuver created saturation artifacts in all unobtrusive sensing modalities (see red (pointed) ellipsoids). It is interesting to note that both reference sensors were influenced as well. The influence was obvious for the respiratory reference, where a mouthpiece was used that the users had to release during stand up/sit down and the head-torsion maneuver. However, even the standard ECG using adhesive electrodes was disturbed by strong motion artifacts.

The influence of motion on the PPG sensor was highly dependent on the direction of motion, see blue (dashed) ellipsoids. Here, a shift of the torso to the right (towards the sensor) created a much smaller artifact than a shift away from the sensor. The same applied to the torso torsion maneuver, where a clockwise rotation increased pressure on the right side of the subject’s back and thus created a better contact with the sensor, while a counterclockwise rotation induced a strong artifact. This was particularly obvious in the high-amplitude case, where the sensor was not covered by the subject anymore.

**Figure 8:**
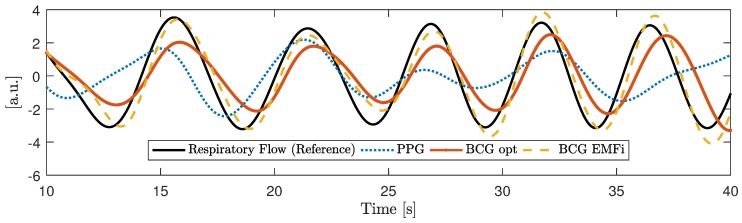
Excerpt of the measurement presented in Figure 7 to highlight the respiratory component. All signals were bandpass filtered with a passband of 0.1 to 0.3 Hz. The PPG signal was inverted to better align its phases for visual comparison.

It is also worth noting that the PPG and both BCG signals showed a strong respiratory component, which is highlighted in Figure 8.

Finally, the tracking of the head marker resulted in the desired behavior. First, the direction of movement could be inferred from the sign of the data and was consistent with the protocol. Moreover, a qualitative difference in amplitude between the first set (low amplitude, t=60,…,270 s) and the second set (high amplitude, t=300,…,510 s) could clearly be seen.

### 3.2. Motion Artifact Analysis

**Figure 9:**
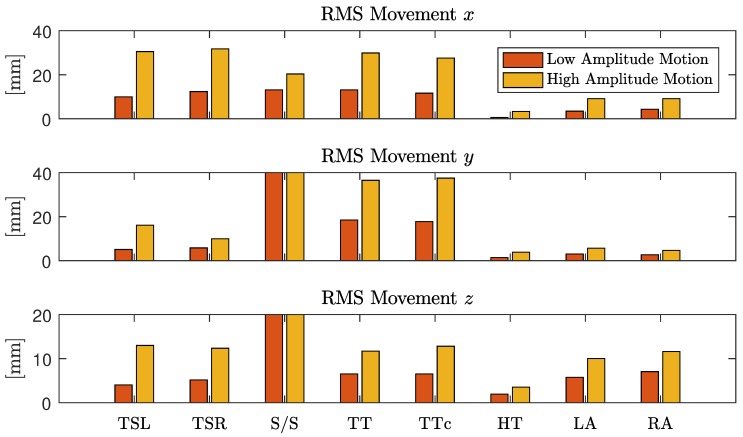
Motion amplitude for individual maneuvers averaged over all subjects.

In Figure 9, the results of the motion quantification averaged over all subjects are displayed. First, differences between the low and the high amplitude scenario were obvious and confirm that the instructions given to the subjects resulted in the desired amplitude variation. Moreover, differences between individual maneuvers could be observed. Shift and torsion of the thorax had a similar influence on the movement in the *x*-direction (left-right axis), while torsion had a much larger influence on the *y*-direction (dorsoventral axis). As expected, torsion of the head only had a minimal influence on the motion of the thorax.

**Figure 10:**
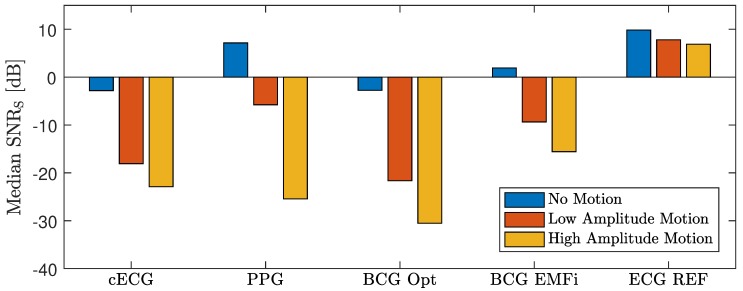
Median ${\mathrm{SNR}}_{S}$ for all subjects and maneuvers for the three scenarios “no motion”, “low amplitude motion” and “high amplitude motion”.

Figure 10 displays the median ${\mathrm{SNR}}_{S}$ for all subjects separated into artifact-free episodes, the low and the high amplitude scenario. As expected, the reference ECG showed the highest ${\mathrm{SNR}}_{S}$ when compared to the unobtrusive modalities. It is indicated in Figure 7 that both reference and unobtrusive signals deteriorated when the subject moves. Figure 10 demonstrates that the influence of motion and the signal quality at rest were strongly dependent on the sensing modality. From the unobtrusive modalities, only PPG and BCG EMFi exhibited a positive ${\mathrm{SNR}}_{S}$ at rest, indicating that, averaged over all subjects, cECG and BCG Opt did not exhibit a very regular waveform. In the high amplitude scenario, ${\mathrm{SNR}}_{S}$ dropped below −15 dB for all modalities. For cECG and BCG Opt, even the low amplitude motions resulted in a ${\mathrm{SNR}}_{S}$ below this value. For PPG and BCG EMFi, however, small motions only resulted in a reduction to −5.8 dB and −9.4 dB, respectively.

In Table 3, the correlation coefficient between ${\mathrm{SNR}}_{S}$ and motion amplitude is presented for all modalities. As expected, a negative correlation was observed for all modalities and coordinate axis. The values of correlation for cECG and ECG REF were relatively low, while they were highest for BCG EMFi and PPG. In addition, differences between coordinate axes were observed.

In Table 4, the absolute interval estimation error and the coverage is reported for the individual analysis of all unobtrusive modalities and the fusion of all available signals with and without the adaptive prior.

For one, distinct differences between individual modalities existed. On average, PPG and BCG EMFi showed the highest coverage and the lowest error. This is consistent with the observation that these modalities exhibited the highest ${\mathrm{SNR}}_{S}$ in the scenarios “no movement” and “low amplitude movement”. If all modalities were fused, the mean absolute error was 21.0 ms, slightly higher than the optimal error (PPG, 15.6 ms). At the same time, the coverage was improved to 78.9%, much higher than the optimal coverage achieved by a single sensor (BCG EMFi, 63.3%). These values were further improved towards 18.0 ms and 81.6% when the adaptive prior as described in [6] was implemented.

For another, estimation errors differed largely from one subject to the next. This was most pronounced for cECG, where both the smallest (ID 7) and the tallest (ID 4) subject generated an estimation error more than four times the average value. In the case of subject 7, this was even higher for PPG, where the error was more than six times the average. For subject ID 3, the values for cECG and BCG EMFi were in the normal range, whereas the values for PPG and in particular BCG Opt could be considered outliers. Since both sensors were located at the same height, one could speculate if contact pressure was low in this area due to a combination of body height and/or posture.

**Figure 11:**
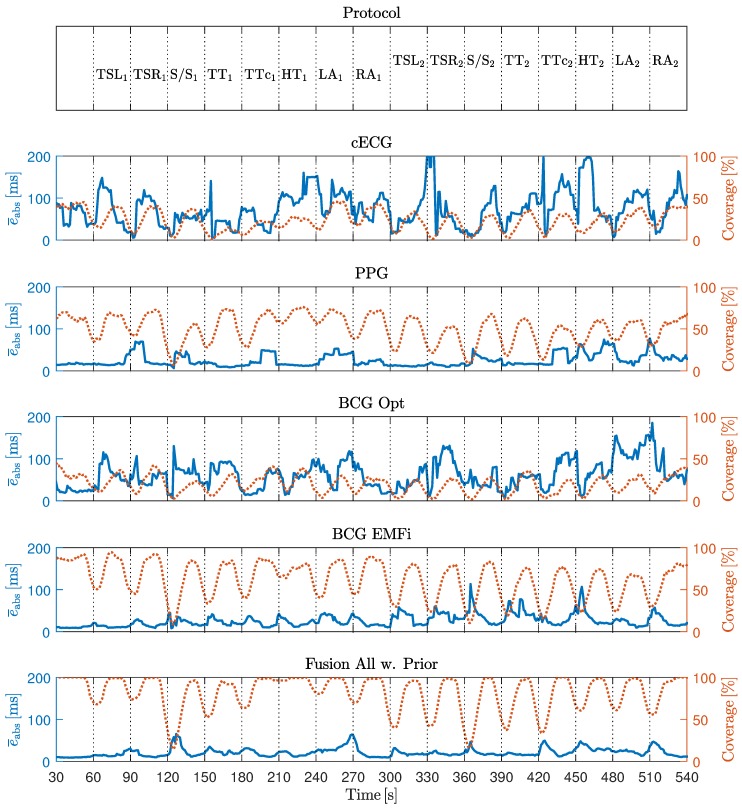
Mean absolute estimation error and coverage, averaged over all subjects in a moving window of 12 s duration. Troughs in the coverage can be observed for all sensors when the motion protocol is executed.

Figure 11 shows the timecourse of coverage and error, averaged over all subjects. Most notably, coverage was reduced when the motion artifacts were present and drops close to zero for the stand up/sit down maneuver, i.e., xCLIE did not detect any beats at all. Moreover, drops in the coverage curves were more pronounced in the second half of the measurement, i.e., in the high amplitude movement scenario. At the same time, no systematic increase in the error curve could be detected. Thus, in Table 5, the correlation of motion and absolute error as well as motion and coverage is displayed. It confirms that a negative correlation between coverage in motion existed for all modalities and coordinates axes. In the fusion scenario, the observed correlations had the largest absolute value. On the other hand, the correlation between motion and estimation error was close to zero. This finding demonstrates that xCLIE rather rejects an interval estimation than reporting a false one.

### 3.3. Video Sequence

**Figure 12:**
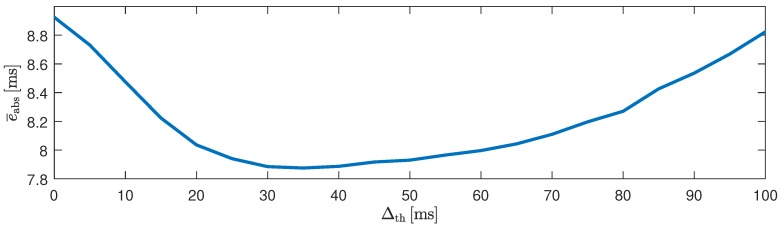
Influence of the parameter $\Delta_{\mathrm{th}}$ on the absolute interval estimation error ${\bar{e}}_{\mathrm{abs}}$. A threshold of $\Delta_{\mathrm{th}}=0$ means using only xCLIE, $\Delta_{\mathrm{th}}\to \infty$ means only intervals obtained via P&T applied to cECG are chosen. In the final implementation, $\Delta_{\mathrm{th}}=40$ ms was used.

**Figure 13:**
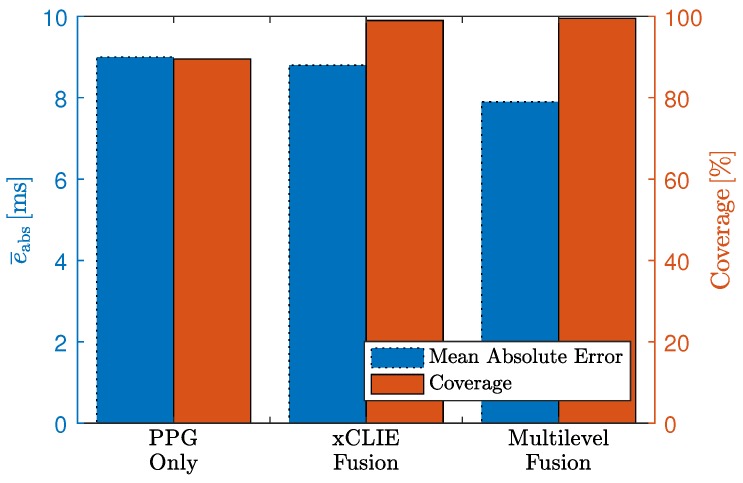
Comparison of xCLIE and multilevel fusion. PPG is listed as best performing individual modality in terms of error and coverage.

In Table 6, the results for the video sequence in terms of absolute beat-to-beat interval estimation error and coverage are reported for all individual modalities and fusion with and without prior. Based on the observations made in the artifact scenario, the sensor BCG Opt was excluded from the fusion process. Similar to the results reported above, the highest coverage (99.0%) was achieved when fusing multiple modalities. Moreover, the error of 8.8 ms was even slightly lower than that of the PPG sensor (9.0 ms) and below the sampling time of 10 ms. It is interesting to note that interindividual differences existed. For one, the error for subjects 7 and 11 were comparatively large. In addition, while the cECG exhibited relatively high error and low coverage on average, it exhibited an excellent performance for subjects two and four. Based on these observations, the algorithm described in Section 2.3 was developed and optimized. In Figure 12, the influence of the parameter $\Delta_{\mathrm{th}}$ is shown. Using the proposed approach with $\Delta_{\mathrm{th}}=40$ ms, the estimation error was reduced to 7.9 ms, with the coverage of the “fusion w. prior” approach of 99.5%. In total, 21,151 (approximately 74%) of intervals estimated by xCLIE were replaced by detections made by the P&T algorithm on the cECG channel. The results are visualized in Figure 13.

## 4. Discussion and Outlook

“MuSeSe”, the multisensor armchair, demonstrates that accurate and unobtrusive health monitoring is possible by combining several sensing modalities. Moreover, the use of a high-quality motion capture system allows a detailed analysis of motion artifacts. With respect to the physical and abstraction layer, several observations are discussed in the following.

First, the integration of multiple sensors improved the overall estimation quality. However, while the optical BCG sensor was able to recover some cardiac information, the results were inferior to any other modality. Even worse, its inclusion into the fusion process resulted in so-called “catastrophic fusion”, i.e., a reduction in estimator quality and it was thus excluded. Since the sensor was proven to be comparable to the EMFi mat in previous works [5], it is likely that its position in this setup was suboptimal. For one, the BCG is known to have the strongest component in the craniocaudal axis, i.e., in the direction of the EMFi mat in the seat. It was shown in measurements performed in sleep laboratories that good signals can be achieved by placing the mat under lying subjects [4], which means that a dorsoventral BCG component was captured. It can be expected that the contact pressure is much higher in such a scenario, which would explain the inferior signal quality in this setup. Thus, while the inclusion of multiple sensors and subsequent sensor fusion is beneficial for unobtrusive health monitoring in general, sensor type and location have to be chosen with care.

Two subjects, 7 and 11, exhibited notable inferior results in terms of beat-to-beat interval estimation in the video sequence. While subject 7 was the only female volunteer, another difference to the other subjects was her height. While mean and SD were 181 cm and 8.95 cm respectively, subject 7 was only 161 cm tall. Thus, all sensors except BCG EMFi had a different relative position for this subject. Accordingly, cECG and PPG exhibited particularly bad results, which is consistent with the observations made in the motion artifact sequence. In the future, the incorporation of multiple sensors that accommodate for different body heights could help to make the setup more suitable for a greater variety of users. Interestingly, while cECG showed an inferior performance for the tallest subject (ID 4) in the motion artifact sequence, the opposite could be observed during the video sequence. While not an encouraging observation per se, it is consistent with reports that a multitude of factors, ranging from worn fabric over humidity to contact pressure, influence the quality of cECG [10,13,30,31].

As to subject 11, the most distinct difference here was the age of 71 years compared to the mean (SD) of 33.54 (12.04). Moreover, visual inspection of the reference ECG revealed several ectopic beats, which might be the cause for the increased mean absolute error, as a change in beat morphology could be observed in the unobtrusive signals. Since ectopic beats were only present in one subject, no further analysis was performed. In the future, a larger study including different ages and medical conditions should analyze the detection of, for example, extrasystoles automatically. Results presented in [32] show that, at least with invasive modalities, features derived from xCLIE may be used in intelligent alarming.

In the presented analysis, the shape-based SNR reported results that were consistent with those of xCLIE. This is not surprising, as xCLIE is based on self-similarity, which is indirectly quantified by ${\mathrm{SNR}}_{S}$. Consistently, a high correlation of motion and coverage could be established. At the same time, a high negative correlation of ${\mathrm{SNR}}_{S}$ and the motion amplitude as determined by the motion capture system could be observed. Thus, the combination of motion capture and ${\mathrm{SNR}}_{S}$ can be a powerful tool for the analysis of unobtrusive sensing modalities. For example, the relatively small negative correlation between ${\mathrm{SNR}}_{S}$ and motion amplitude for cECG can be explained by the fact that even small motions can cause nonlinear saturation effects, which also render any further analysis impossible. On the other hand, the small negative correlation between motion and the ${\mathrm{SNR}}_{S}$ of the reference ECG using adhesive electrodes is attributed to its relative robustness. Finally, PPG and BCG EMFi exhibit a comparatively large negative correlation, indicating that, combined with the observation that the ${\mathrm{SNR}}_{S}$ during no motion is relatively high, smaller motions have a smaller impact and might be compensable. Similarly, a very low ${\mathrm{SNR}}_{S}$ below −20 dB for the optical BCG sensor in the backrest even for small motion amplitudes was consistent with the finding that an exclusion of the sensor improved overall results. On the other hand, for this specific setup, motion amplitudes in the sub-centimeter range could be compensated, especially when multiple modalities were fused. Finally, the expected deterioration of the reference ECG with motion could also be observed, indicating that the presented methodology might be useful for the analysis of other health monitoring modalities, ranging from standard ECG to wearable sensors.

The ${\mathrm{SNR}}_{S}$ approach presented here was designed for the offline analysis and quantification of unobtrusive modalities and motion artifacts. By design, a reference segmentation needs to be available and thus it is obvious that the metric cannot be used to determine the signal quality when recording only unobtrusive signals. If one wanted to use the ${\mathrm{SNR}}_{S}$ metric for this, an approach that estimates both template and segmentation iteratively is conceivable but would need extensive analysis and validation. Another drawback of the ${\mathrm{SNR}}_{S}$ approach as presented here is that heartbeats with severely irregular waveforms will also result in a lower value. However, as argued above, a purely frequency-range based evaluation has drawbacks that might be considered even more severe. In addition, if the database is large enough, a template for specific irregular beats could be extracted when the signal is undisturbed, and then used for evaluation when the signal exhibits both motion artifacts and irregular cardiac activity. Again, a reference segmentation and an additional beat annotation is necessary and limits the approach to offline analysis.

Combining xCLIE fusion and QRS detection on the capacitively coupled ECG improved the overall results of beat-to-beat interval estimation. In other words, cECG is the single most accurate unobtrusive source to localize heartbeats, if the signal quality is high enough. This demonstrates the importance of this modality and research in the area to further improve its reliability. Finally, while the algorithm did not exhibit a great sensitivity with respect to the heuristic parameter $\Delta_{\mathrm{th}}$, a systematic approach to optimize it should be investigated.

## 5. Conclusions

In this work, methods for motion artifact quantification and sensor fusion were presented for an unobtrusive health monitoring scenario. A multimodal sensor setup integrated into an armchair was presented that combined capacitively coupled electrocardiography, reflective photoplethysmography, two high-frequency impedance sensors and two types of ballistocardiography sensors. To quantify motion artifacts, a motion protocol was performed by nine healthy volunteers. As reference for motion and cardiorespiratory activity, a motion capture system and reference sensors were employed. To analyze the effect of motion on different modalities quantitatively, the shape-based signal-to-noise ratio SNR${}_{S}$ was introduced. It could be shown that this measure correlated well with the coverage of beat-to-beat interval estimation. Based on the motion analysis, an optimal combination of sensors and a mixed-level fusion methodology was developed and evaluated. Using the proposed approach, beat-to-beat heart-rate was estimated with a coverage of 99.5% and a mean absolute error of 7.9 ms on 425 min of data from seven volunteers in a proof-of-concept measurement scenario. In the future, the presented approach can be used for general analysis and optimization of unobtrusive health monitoring setups.

## Figures and Tables

**Table 1:** Details on subjects who volunteered for the measurements.

Subject	Age	Height	Weight	BMI	Sex	Scenario		Duration
ID	[years]	[cm]	[kg]	[kg/m${}^{2}$]		Motion	Video	[min]
1	32	188	78	22.07	M	x		
2	31	185	91	26.59	M	x	x	121
3	30	182	81	24.45	M	x	x	72
4	34	195	118	31.03	M	x	x	60
5	27	179	70	21.85	M	x		
6	32	169	75	26.26	M	x	x	51
7	28	161	56	21.60	F	x	x	45
8	29	178	65	20.52	M	x		
9	28	188	82	23.20	M	x		
10	27	183	70	20.90	M		x	39
11	71	184	101	29.68	M		x	36
Mean	33.55	181.09	80.59	24.38	∑F=1	∑=9	∑=7	60.57
SD	12.04	8.95	16.56	3.42	∑M=10			27.22

**Table 2:** Motion protocol. Amplitude values were given as instructions to the subject. No amplitude variation was performed for stand up/sit down maneuver. Modified from [22].

Acronym	Description	Amplitude${}_{1}$	Amplitude${}_{2}$
TSL	Torso Shift Left	5 cm	10 cm
TSR	Torso Shift Right	5 cm	10 cm
S/S	Stand Up/Sit Down	n.a.	n.a.
TT	Torso Torsion Clockwise	10${}^{\circ }$	20${}^{\circ }$
TTc	Torso Torsion Countercl.	10${}^{\circ }$	20${}^{\circ }$
HT	Head Torsion	45${}^{\circ }$	70${}^{\circ }$
LA	Lift Left Arm	Breast Height	Shoulder Height
RA	Lift Right Arm	Breast Height	Shoulder Height

**Table 3:** Correlation coefficient between ${\mathrm{SNR}}_{S}$ and the RMS motion with respect to the *x*, *y*, and *z* coordinates.

Modality	$\rho_{{\mathrm{SNR}}_{S},\mathrm{motion}}$		
	*x*	*y*	*z*
cECG	−0.33	−0.22	−0.15
PPG	−0.69	−0.67	−0.52
BCG Opt	−0.65	−0.64	−0.55
BCG EMFi	−0.67	−0.72	−0.68
ECG REF	−0.25	−0.33	−0.24

**Table 4:** Mean absolute interval estimation error (${\bar{e}}_{\mathrm{abs}}$) in milliseconds and coverage in percent for all subjects in the motion artifact scenario.

Subject ID	${\bar{e}}_{\mathrm{abs}}$ [ms] (Coverage [%])											
	cECG		PPG		BCG		BCG		Fusion		Fusion	
					Opt		EMFi		All		All w. Prior	
1	67.4	(7.5)	12.5	(70.4)	60.0	(25.5)	9.6	(73.3)	10.0	(77.8)	9.1	(80.7)
2	13.8	(62.4)	11.2	(82.8)	32.6	(36.7)	22.9	(74.7)	9.6	(86.4)	11.0	(87.8)
3	12.3	(59.0)	32.7	(20.6)	115.4	(12.1)	22.8	(63.1)	15.6	(79.7)	13.7	(82.1)
4	162.8	(12.9)	9.5	(79.4)	30.7	(30.0)	20.6	(55.1)	12.8	(78.8)	13.7	(81.1)
5	44.6	(30.0)	15.3	(61.8)	49.1	(14.3)	16.6	(73.9)	24.4	(82.8)	20.4	(84.9)
6	19.9	(26.6)	18.6	(39.4)	16.1	(33.6)	14.5	(69.5)	19.3	(81.5)	16.4	(83.5)
7	180.8	(7.3)	99.2	(8.5)	35.8	(12.9)	39.8	(34.6)	65.9	(67.4)	47.9	(74.1)
8	81.0	(9.2)	16.3	(53.6)	87.6	(12.1)	23.5	(62.6)	23.6	(78.3)	21.2	(81.1)
9	60.0	(7.0)	16.8	(37.2)	64.8	(17.0)	19.2	(56.1)	22.9	(74.9)	16.9	(77.7)
**Gross Average**	37.6	(25.3)	15.6	(52.1)	47.1	(21.6)	19.6	(63.3)	21.0	(78.9)	18.0	(81.6)

**Table 5:** Correlation coefficient between motion and estimation error as well as motion and coverage for all modalities and coordinate axes.

Modality	$\rho_{{\bar{e}}_{\mathrm{abs}},\mathrm{motion}}$			$\rho_{\mathrm{coverage},\mathrm{motion}}$		
	*x*	*y*	*z*	*x*	*y*	*z*
cECG	−0.04	−0.09	−0.06	−0.23	−0.17	−0.16
PPG	−0.05	−0.02	−0.03	−0.30	−0.27	−0.22
BCG Opt	−0.04	−0.05	−0.03	−0.29	−0.18	−0.17
BCG EMFi	0.06	0.10	0.13	−0.47	−0.37	−0.34
Fusion All w. Prior	0.06	0.11	0.11	−0.67	−0.60	−0.53

**Table 6:** Mean absolute interval estimation error (${\bar{e}}_{\mathrm{abs}}$) in milliseconds and coverage in percent for all subjects in the video scenario.

Subject ID	${\bar{e}}_{\mathrm{abs}}$ [ms] (Coverage [%])											
	cECG		PPG		BCG		BCG		Fusion		Fusion	
					Opt		EMFi				w. Prior	
2	5.5	(96.5)	6.6	(99.5)	38.4	(37.3)	7.6	(98.6)	4.4	(99.7)	4.5	(99.8)
3	9.3	(80.1)	12.6	(89.3)	63.9	(24.5)	20.6	(71.8)	8.4	(98.9)	8.9	(99.6)
4	4.3	(96.5)	7.6	(99.7)	45.5	(29.1)	20.4	(62.6)	5.8	(99.0)	6.0	(99.1)
6	11.1	(78.5)	6.8	(99.5)	20.9	(57.9)	17.0	(71.6)	5.5	(99.9)	5.8	(99.8)
7	46.1	(34.3)	21.2	(37.1)	26.5	(50.9)	27.8	(72.2)	24.1	(97.8)	20.8	(98.8)
10	21.1	(52.0)	10.0	(90.0)	37.4	(28.3)	10.0	(89.9)	7.8	(97.8)	7.3	(99.2)
11	74.8	(25.6)	9.0	(83.2)	180.9	(8.1)	12.4	(89.2)	18.2	(97.8)	22.0	(99.1)
**Gross Average**	11.3	(75.7)	9.0	(89.5)	40.8	(33.9)	14.3	(81.8)	8.8	(99.0)	8.9	(99.5)

## Abbreviations

The following abbreviations are used in this manuscript:

BCG	ballistocardiography
BMI	body–mass–index
cECG	capacitively coupled electrocardiography
CLIE	continuous local interval estimator
DAQ	data acquisition system
DRL	driven-right-leg
ECG	electrocardiography
EMFi	electromechanical film
PPG	photoplethysmography
P&T	Pan and Tompkins
RC	resistor-capacitor
RMS	root mean square
SD	standard deviation
SNR	signal-to-noise ratio
SNR${}_{S}$	shape-based signal-to-noise ratio
